# Median Nerve Compression in Carpal Tunnel Caused by a Giant Lipoma

**DOI:** 10.1155/2014/654934

**Published:** 2014-05-04

**Authors:** F. Fazilleau, T. Williams, J. Richou, V. Sauleau, D. Le Nen

**Affiliations:** ^1^Department of Orthopaedic Surgery, CHRU La Cavale Blanche, Brest Hospital, Boulevard Tanguy Prigent, 29200 Brest, France; ^2^Faculty of Medecine Brest, University of Western Brittany, 22 Rue Camille-Desmoulins, 29200 Brest, France; ^3^Orthopaedic Unit, Clinic of Ter, Chemin de Kerbernès, 56270 Lorient, France; ^4^Orthopaedic Unit, Clinic Saint Michel and Sainte Anne, 88 Rue de Kergéstin, 29000 Quimper, France

## Abstract

A lipoma is a common, benign soft-tissue tumor that rarely arises in the upper limb. When one does occur in the hand, the location of the lipoma can cause nerve compression, which can mimic carpal tunnel symptoms. Magnetic resonance imaging is the visualization modality of choice for diagnosis and surgical planning of lipomas. Surgical resection is recommended to relieve the neurological manifestations of this disease. The surgeon should always suspect liposarcoma first before voluminous, atypical, or recurrent tumors are considered.

## 1. Introduction

Lipomas are the most common benign tumors found in limbs [1]. Although the occurrence of lipomas in the hand remains rare, between 1 and 3.8% of benign tumors in hands [2], their clinical and therapeutic particulars require a physician to consider each case specifically. The rich neurovascular environment and weak compliance of the soft tissue in hands are the foundations for this specificity. Indeed, the lack of dead spaces in the hand compartments causes, from the beginning of tumor expansion, a fast compression of the neurovascular endings. This compression produces an atypical clinical presentation that can mimic carpal tunnel syndrome. In the literature, “giant lipoma” is defined as a lesion that measures more than 5 cm [3]. Using a case report to support our claims, we provide information about lipomas in the hand and the diagnostics traps that they constitute.

## 2. Case Report

A 69-year-old right-handed woman presented with a gradual tumefaction on the palm of her right hand that developed without any traumatic or microtraumatic antecedent.

Interrogation of the patient found a progressive evolution over several months with gradual acroparesthesia in the median nerve territory associated with palmar pain. Physical examination discovered a decrease in epicritic sensitivity with the Weber test, without evidence for motricity impairment, using preserved prehensile strength and digital winding as readouts.

Palpation revealed limited renitent tumefaction on the palm of the hand, without cutaneous suffering. There were no signs of septic diseases (fever, regional adenopathy, lymphangitis, or cutaneous inflammation) or tumoral dissemination (asthenia, weight loss, or alteration in the global condition).

**Figure 1:**
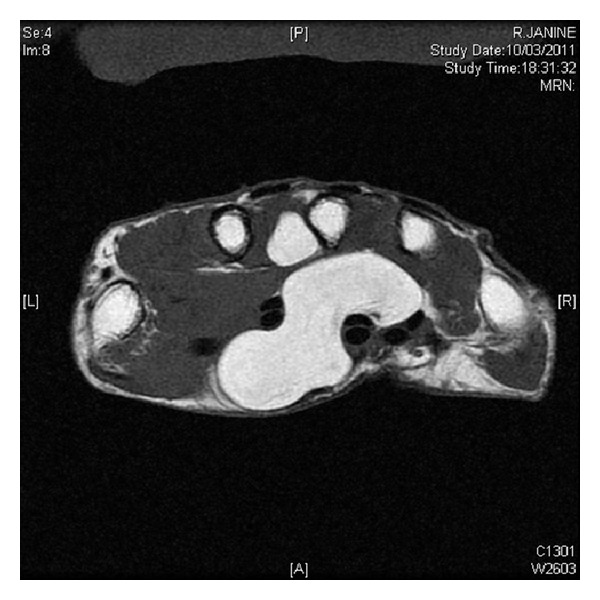
MRI axial view showing the tumor with spontaneous hypersignal rejecting flexors tendons without invasion.

**Figure 2:**
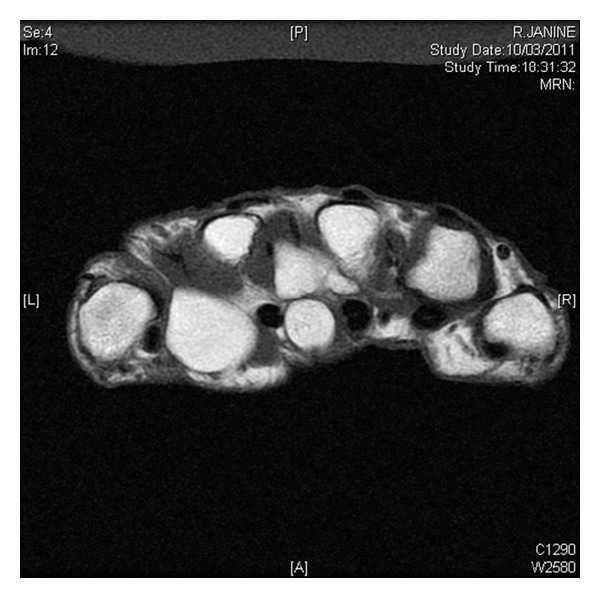
MRI axial view showing distal extension of lipoma in the first metacarpal space.

**Figure 3:**
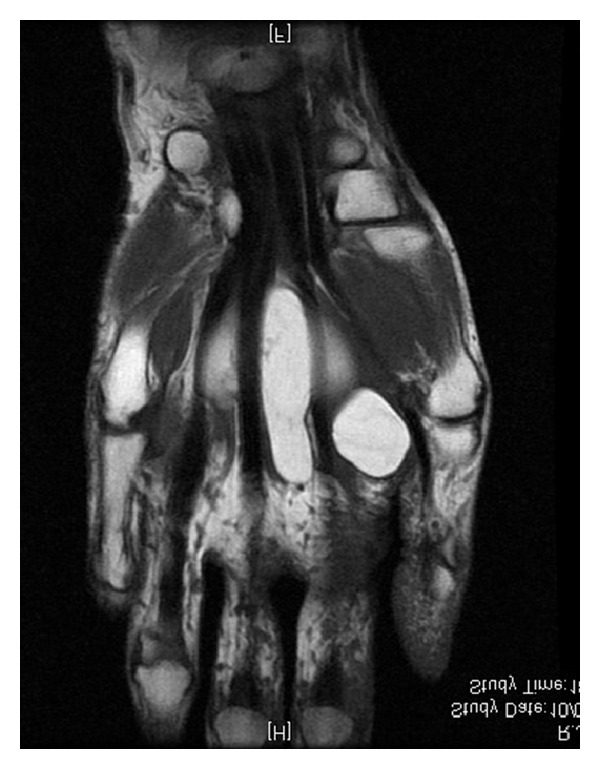
MRI frontal view showing lipoma among flexors tendons and extending to the carpal tunnel.

**Figure 4:**
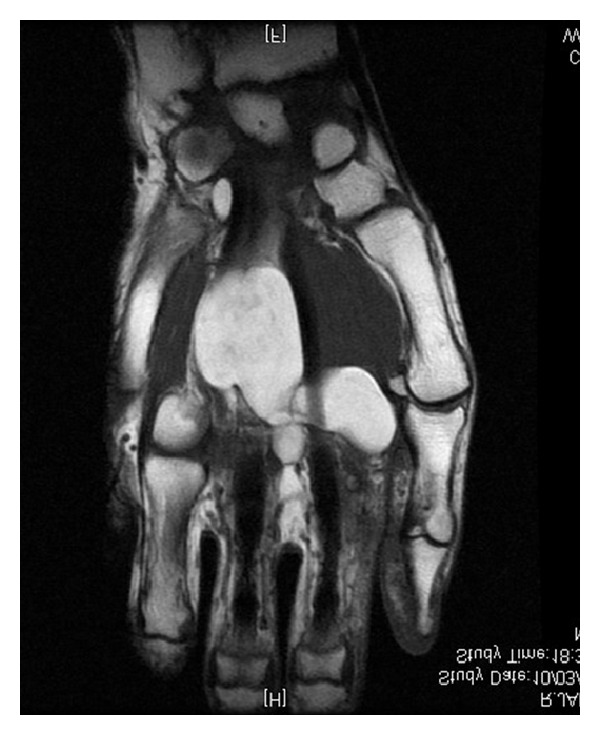
MRI frontal view showing tumor's infiltration without invasion.

Functional exploration with electromyography identified a sensitive injury downstream of the carpal tunnel. Standard X-rays did not found abnormalities. Magnetic resonance imaging (MRI) found a large tumor, which was homogeneous, multilobulated, and with a sharp border. The tumor had an intense hypersignal in T1 and a poor fat suppression signal (Figures 1 and 2). The lesion was located between the thumb and the fourth intermetacarpal space, invading the totality of the palm of the hand and extending into each interosseous space (Figures 3 and 4).

Resection was performed under locoregional anesthesia with an axillary block. The patient was arranged in a dorsal decubitus position; a pneumatical tourniquet was placed around the arm and inflation was maintained during the surgical procedure.

**Figure 5:**
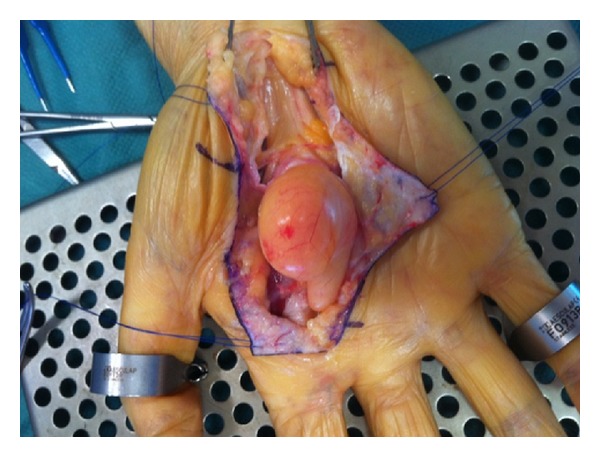
Peroperating view showing lipoma with fat aspect and well-defined border.

**Figure 6:**
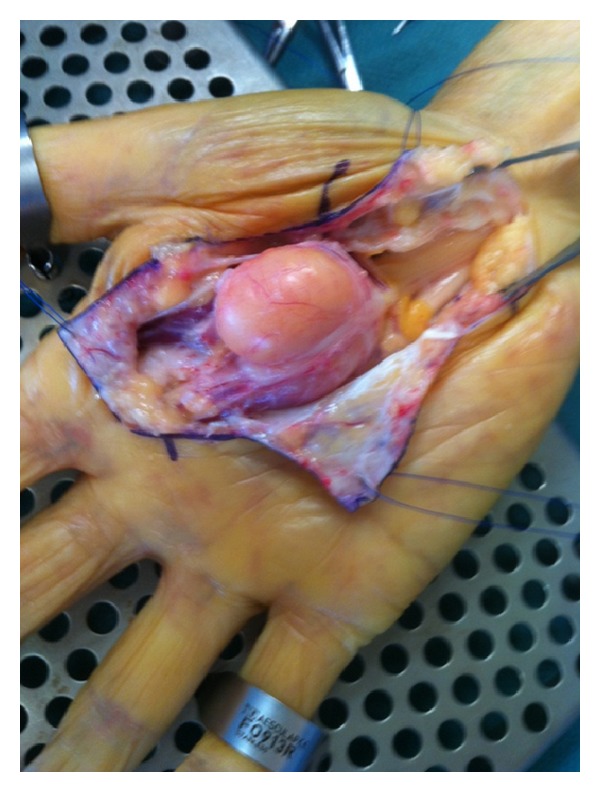
Peroperating view showing intimate relation between lipoma and nervous structures.

**Figure 7:**
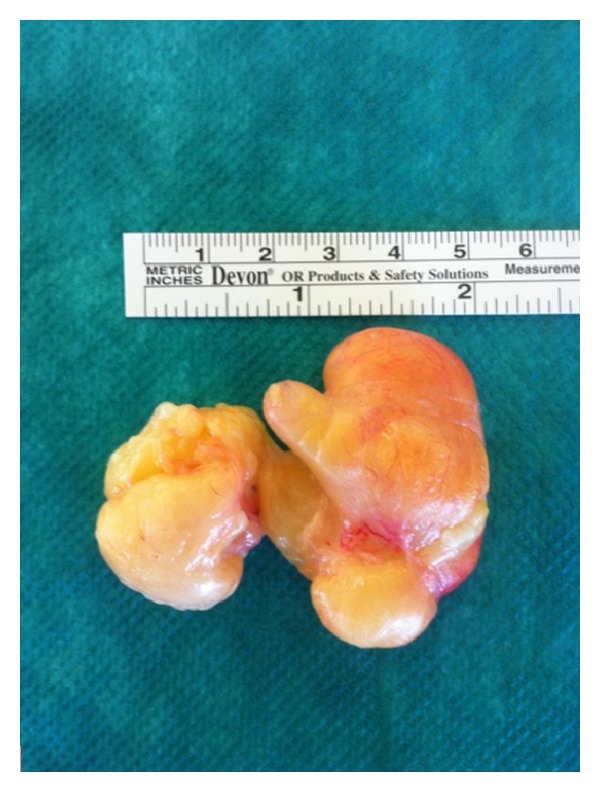
Operating piece after excision.

A palmar zigzag approach through palmar-crease was chosen face up of the tumor to promote release of the neurovascular pedicle joining the tumor to the hand (Figure 5). Lipoma was easily exposed after skin incision and opening of the mid-palmer fascia. The ulnar neurovascular pedicle of the index finger was flattened and repulsed by the lesion (Figure 6). The tumor was then removed in one unique piece despite of its bilobate appearance. Lipoma had a first bulge immediately under the skin and a deeper second one developed in the first intermetacarpal space. Its two parts were joined by a bridge passing between the flexor tendons of the second and the third fingers (Figure 7). Dissection was facilitated by noninvasive nature of the lesion with well-defined border. Particular attention was paid upon release of the pedicles. After excision, the skin was closed with nonabsorbable sutures.

Postoperatively, the patient went home with scar care every other day and a prophylactic treatment for algoneurodystrophy with vitamin C for one month. No immobilization was used.

Complete healing occurred after 14 days. The last clinical review at the one-month postoperative clinical examination showed entire disappearance of the acroparesthesia and total functional recovery including full mobility of the fingers, absence of pain, and restoration of the grip strength. No specific rehabilitation was necessary.

Histopathological analysis concluded that the lesion was a lipocytic lipoma with no evidence of malignancy.

## 3. Discussion

Lipomas represent 16% of all mesenchymal tumors [4] and are frequently the first type of benign tumor to occur in limbs. Although they are predominantly localized on the lower limbs, their localization in the hand is particularly interesting because of their atypical expression there.

Lipomas of the hand principally occur in individuals between 50 and 60 years of age [3, 4]. Patients usually report progressive development of a soft, mobile, and painless tumefaction on one of their hands. Its classical evolution is marked by emergence of a mass syndrome. The appearance of pain is often the result of nerve compression, which can occur at different locations.

Its clinical expression is identified by the topography of the compression: carpal tunnel syndrome in the case of median nerve compression [3, 5], hypoesthesia of the half pulp in the case of digital collateral nerve compression [4], and ulnar hypoesthesia in the case of Guyon's canal compression [6]. Other localizations are more anecdotal: compression of the superficial sensory branch of the radial nerve in the anatomical snuffbox [2] or compression of the posterior interosseous nerve [7]. We have found no evidence for any symptomatic vascular compression in the literature. Indeed, there are no apparent links between the tumor volume and the patient's symptomatology [8].

Generally, standard X-ray examinations do not aid in the diagnosis because they only show a homogeneous opacity in the soft tissue. However, sometimes they reveal calcifications in the tumoral body or a cortical bone condensation, in the case of parosteal lipoma [9].

Ultrasound is an effective tool to explore any mass existence in the hand. Physicians easily request ultrasound in case of atypical symptoms of carpal tunnel syndrome. Ultrasound provides a view of the anatomy of nerve as well as of surrounding structures and allows real-time and painless assessment of median nerve entrapment. Typical findings associated with nerve compression include enlargement of the nerve proximal to the site of compression, decreased echogenicity, and increased vascularity [10]. Moreover ultrasound enables precise differentiation of the space occupying lesions causing median nerve compression [11]. Lipomas classically appear as elongated lesions with their greater diameter parallel to the skin and with a length-anteroposterior diameter ratio of approximately 3 : 1. Lesion usually appears in homogenous hyperechoic structure with well-defined margins and without posterior enhancement or color doppler signal.

MRI is the visualization modality of choice for exploration of tumors of the hand. Primarily, it allows a diagnostic orientation of the tumor, and, secondly, it shows anatomical relationships between the tumor and the neurovascular structures. Classically, lipomas appear as a homogeneous mass, with a sharp border, spontaneous T1 and T2 hypersignals, reduced signal intensity after erasure of the fat signal, and no raising in the signal using the gadolinium contrast agent [12, 13]. Fine cuts, smaller than 2 mm, allow identification of septae inside the tumor, which appear as small, discreetly raised features after gadolinium injection [12]. In a 134-case review of hand and wrist tumors, Capelastegui compared MRI examinations with histological results. He concluded that MRI has a positive predictive value, near 94% [1].

We advocate for a thorough radiographic examination in all cases of atypical clinical presentation of carpal tunnel syndrome including unilateral symptoms, sudden onset, unusually young patient, and clinical mass syndrome.

Differential diagnosis for this type of tumours includes principally fibrolipoma of the median nerve [14] and low grade liposarcoma. Fibrolipoma of the median nerve represents only 2% of tumors of the soft tissue [14]. With MRI, it appears as a well-differentiated mass, with a sharp border and heterogeneous signal consisting of a mixed composition of fat and fibrosis. A pathognomonic sign for this disease is tumefaction of the fat signal crossed by nerve fibers with a low signal [15]. It is a benign tumor that develops from fibroblasts and adipocytes of the epineurium. It always remains purely intraneural [16]. Therapeutic support is difficult because it is often impossible to dissociate the tumor and the regular nerve fibers with resection.

Liposarcoma remains the most prevalent diagnosis [17], particularly in cases of recurrent lipoma [18]. Although it is mainly found in a well-differentiated form in 40–45% of cases [17], all forms can be found. MRI can provide important insights into this disease. Liposarcoma appears as a heterogeneous mass, wrong limited, and with a fuzzy border. Analysis of the features in the body of the liposarcoma reveals thickened elements, which are raised after gadolinium injection, that correspond to muscles fibers [1, 13]. Evolution of the disease results in local recurrence and local aggressive extension. Risk is correlated with the level of cellular differentiation that reflects the anaplastic potential [17, 19].

Rarely, other differential diagnoses occur and could be another tumor of the soft tissue, such as a ganglion cyst or giant-cell tumor [9]. Leiomyoma is an uncommon cause of carpal tunnel syndrome resulting from space occupying lesion. It appears that leiomyoma preferentially affects a young male population like that described by Chalidis et al. [20].

The size of 5 cm is the classical cutoff for definition of giant lipoma [3, 5, 8, 21, 22]. This definition is based on the infrequency of lipomas exceeding this size. Tumor more than 5 cm should always raise the suspicion of malignancy. Myhre-Jensen in a clinical review of 1331 benign soft tumors including 640 lipomas found a tumor size less than 5 cm in 95 percent of cases. In the same time, more than 50 percent of the 72 malignant soft tissue sarcomas diagnosed during the study measure 5 cm or more [21]. Similarly, Rydholm and Berg in a retrospective analysis of 428 cases of lipomas find a ratio of solitary lipoma to sarcoma to be 150/1 for tumors smaller than 5 cm and 20/1 for tumors of 5 cm or larger [22].

Therapeutically, surgical resection of lipomas is the single treatment that allows liberation of the compressed nerve endings and effectively removes the tumor. Surgeons should perform a monobloc resection and a careful, safe dissection of the neurovascular branches to maximally reduce the risk of iatrogenic lesions. Local recurrences are rare with histological confirmation of the benign nature of the lipoma. However, some authors assert that the risk of recurrence correlates with the degree of heterogeneity found in the first MRI examination [8].

## 4. Conclusion

The typical or atypical manifestation of nerve compressions in the hand should be investigated for a tumor syndrome in a clinical examination. Practitioners should then try to link the tumor localization with the nerve manifestations. Functional exploration with electromyography is indispensable for authentication of the level of compression. Next, MRI is the best morphological procedure, in terms of predictive values, to explore tumors of the hand and wrist. The size of 5 cm should be the limit for definition of a giant lipoma for hands. Moreover, this size is important to define the level beyond which practitioners must first perform a biopsy to exclude the risk of liposarcoma before complete resection. Surgery is the modality of choice to treat such lesions and results in a good functional recovery. The primary risk remains the risk of iatrogenic lesions during surgical resection, caused by the intimate proximity between the lipoma and neurovascular endings.
